# Interspecific interactions between wild felids vary across scales and levels of urbanization

**DOI:** 10.1002/ece3.1812

**Published:** 2015-12-09

**Authors:** Jesse S. Lewis, Larissa L. Bailey, Sue VandeWoude, Kevin R. Crooks

**Affiliations:** ^1^ Department of Fish, Wildlife, and Conservation BiologyGraduate Degree Program in EcologyColorado State UniversityFort CollinsColorado80523; ^2^ Department of Microbiology, Immunology, and PathologyColorado State UniversityFort CollinsColorado80523

**Keywords:** Bobcat, competition, detection probability, *Lynx rufus*, mountain lion, occupancy, *Puma concolor*, residential development, species interactions, urban gradient

## Abstract

Ongoing global landscape change resulting from urbanization is increasingly linked to changes in species distributions and community interactions. However, relatively little is known about how urbanization influences competitive interactions among mammalian carnivores, particularly related to wild felids. We evaluated interspecific interactions between medium‐ and large‐sized carnivores across a gradient of urbanization and multiple scales. Specifically, we investigated spatial and temporal interactions of bobcats and pumas by evaluating circadian activity patterns, broad‐scale seasonal interactions, and fine‐scale daily interactions in wildland–urban interface (WUI), exurban residential development, and wildland habitats. Across levels of urbanization, interspecific interactions were evaluated using two‐species and single‐species occupancy models with data from motion‐activated cameras. As predicted, urbanization increased the opportunity for interspecific interactions between wild felids. Although pumas did not exclude bobcats from areas at broad spatial or temporal scales, bobcats responded behaviorally to the presence of pumas at finer scales, but patterns varied across levels of urbanization. In wildland habitat, bobcats avoided using areas for short temporal periods after a puma visited an area. In contrast, bobcats did not appear to avoid areas that pumas recently visited in landscapes influenced by urbanization (exurban development and WUI habitat). In addition, overlap in circadian activity patterns between bobcats and pumas increased in exurban development compared to wildland habitat. Across study areas, bobcats used sites less frequently as the number of puma photographs increased at a site. Overall, bobcats appear to shape their behavior at fine spatial and temporal scales to reduce encounters with pumas, but residential development can potentially alter these strategies and increase interaction opportunities. We explore three hypotheses to explain our results of how urbanization affected interspecific interactions that consider activity patterns, landscape configuration, and animal scent marking. Altered competitive interactions between animals in urbanized landscapes could potentially increase aggressive encounters and the frequency of disease transmission.

## Introduction

Species interactions have long been recognized as a driving factor in shaping ecological communities and influencing the spatial and temporal distribution of animals (Darwin 1859; Schoener 1974; Carothers and Jaksić 1984). Gause (1934) demonstrated that two species with the same ecological requirements, or niches, could not occupy the same area (i.e., the competitive exclusion principle; Hardin 1960). However, species with seemingly similar ecological requirements can coexist by exploiting different habitat features (e.g., Gause 1934; MacArthur 1958). In addition, two species with apparently different niches can have potentially strong interactions that influence the behavior, demography, and distribution of the subordinate species (Palomares and Caro 1999). Landscape change resulting from anthropogenic factors, such as urbanization, can alter species interactions and ecological communities in human‐modified landscapes, which can have rippling effects throughout the ecosystem (Crooks and Soulé 1999; Faeth et al. 2005); however, this area of research has been relatively understudied until recently (Magle et al. 2012). Given the expansive current human footprint globally (Leu et al. 2008; Schneider et al. 2009; Nickerson et al. 2011) and projected rates of additional extensive landscape change resulting from human development (Theobald 2005; Seto et al. 2011), research on interspecific competition (i.e., between species) should focus on understanding how anthropogenic factors (particularly urbanization) influence species interactions and the resulting ecological implications (Magle et al. 2012). Studies comparing competition across a gradient of urbanization can further our understanding for how anthropogenic factors alter species interactions (McDonnell and Pickett 1990; McDonnell and Hahs 2008).

Urbanization currently covers hundreds of millions of acres globally (Schneider et al. 2009; Nickerson et al. 2011) and is projected to expand by hundreds of millions of acres within the next few decades (Cohen 2003; Theobald 2005; Theobald and Romme 2007; Seto et al. 2011). Different forms of urban development, however, can result in varying landscape pattern and impacts on animals. For example, urban (<0.1 ha [<0.25 acres] per residence) and suburban (0.1–0.68 ha [0.25–1.68 acres] per residence) residential development (Theobald 2005) can create relatively impermeable barriers that alter animal movement. The juxtaposition of residential development with wildland habitat (i.e., primarily natural habitat without human development) creates a wildland–urban interface (WUI), which is often characterized by a linear boundary that can significantly alter ecological processes and populations (Radeloff et al. 2005). Exurban (0.69–16.18 ha [1.69–40 acres] per residence) and rural (>16.18 ha [>40 acres] per residence) residential development (Theobald 2005), which is characterized by low‐density urban development often immersed within natural habitat, might not create barriers and can be permeable to animal movement; human disturbance from these forms of development can pervade the landscape over much broader spatial extents and alter animal behavior and population characteristics (Hansen et al. 2005; Lewis et al. 2015). By influencing animal behavior and demography, all forms of urbanization can potentially alter interactions between species. However, despite the pervasiveness of urbanization and the associated impacts to ecological communities, relatively little is known about how varying levels of urbanization affect interspecific competition for most animals.

Interspecific competition is broadly categorized as either exploitation (resource) or interference (contest) (Birch 1957; Schoener 1983). Exploitation competition occurs when two species indirectly compete using the same resource (e.g., food). Interference competition involves direct (or the potential for direct) interactions, such as fighting, killing, or maintaining a territory. Ultimately, competition can result in spatial and temporal niche partitioning between species, which can occur across fine to broad scales (Schoener 1983; Carothers and Jaksić 1984). For example, sympatric species might segregate spatially across daily or seasonal periods or completely avoid areas used by competitors (Albrecht and Gotelli 2001; Kronfeld‐Schor and Dayan 2003).

Competitive interactions can be particularly strong among sympatric carnivores (Rosenzweig 1966; Palomares and Caro 1999; Creel et al. 2001; Caro and Stoner 2003) and larger species can have substantial competitive effects on subordinate species through asymmetrical competition (Schoener 1983; Persson 1985). Various‐sized carnivores often compete when one species steals or scavenges the food of another species (i.e., kleptoparasitism; Koehler and Hornocker 1991; Gorman et al. 1998; Merkle et al. 2009), which, although potentially rewarding energetically, can be especially risky when subordinate carnivores scavenge on the prey carcasses of larger species. Intraguild predation (i.e., the killing and eating of competitors) and interspecific killing (i.e., the killing of a competitor without consumption) can be powerful expressions of interspecific competition that shapes ecological communities and animal behavior (Polis et al. 1989; Palomares and Caro 1999; Arim and Marquet 2004; Donadio and Buskirk 2006; de Oliveira and Pereira 2014). Ultimately, the threat of aggressive interactions or mortality from interspecific competition can cause subordinate species to use “competition refuges” to avoid dominant species and reduce interspecific competition spatially and temporally (Durant 1998; Berger and Gese 2007).

Within the carnivore community of North America, two species with high potential to interact are the bobcat (*Lynx rufus*) and puma (i.e., mountain lion, cougar, panther; *Puma concolor*), which exhibit broad overlap in their geographic distributions and activity patterns (Koehler and Hornocker 1991; Sunquist and Sunquist 2002; Sánchez‐Cordero et al. 2008; Hass 2009). Bobcats will scavenge on the prey of pumas, thus increasing the opportunity for interspecific interactions, and pumas will kill bobcats (Koehler and Hornocker 1991). In addition, the behavior, movement patterns, and population characteristics of both felids are impacted by human development and disturbance (George and Crooks 2006; Riley et al. 2006, 2010; Beier et al. 2010; Tracey et al. 2013; Wilmers et al. 2013), but to varying degrees (Crooks 2002), which can potentially influence interspecific competition. For example, anthropogenic barriers, such as roadways and urban development, can restrict felid movement patterns (Tracey et al. 2013) and increase space‐use overlap (Riley et al. 2010) and thus influence competitive interactions between these species (Crooks et al. 2010). Urban development can also influence disease transmission; in California, pumas acquired the bobcat strain of feline immunodeficiency virus (FIV), presumably through increased interspecific interactions and encounter rates related to urbanization (Franklin et al. 2007). No studies, however, have explicitly evaluated interspecific interactions between bobcats and pumas to understand how varying levels of urbanization influence wild felid interactions. Such evaluations would provide important information about altered competitive interactions, interspecific killing between animals, and the potential for novel modes of disease transmission across urbanizing landscapes.

**Figure 1:**
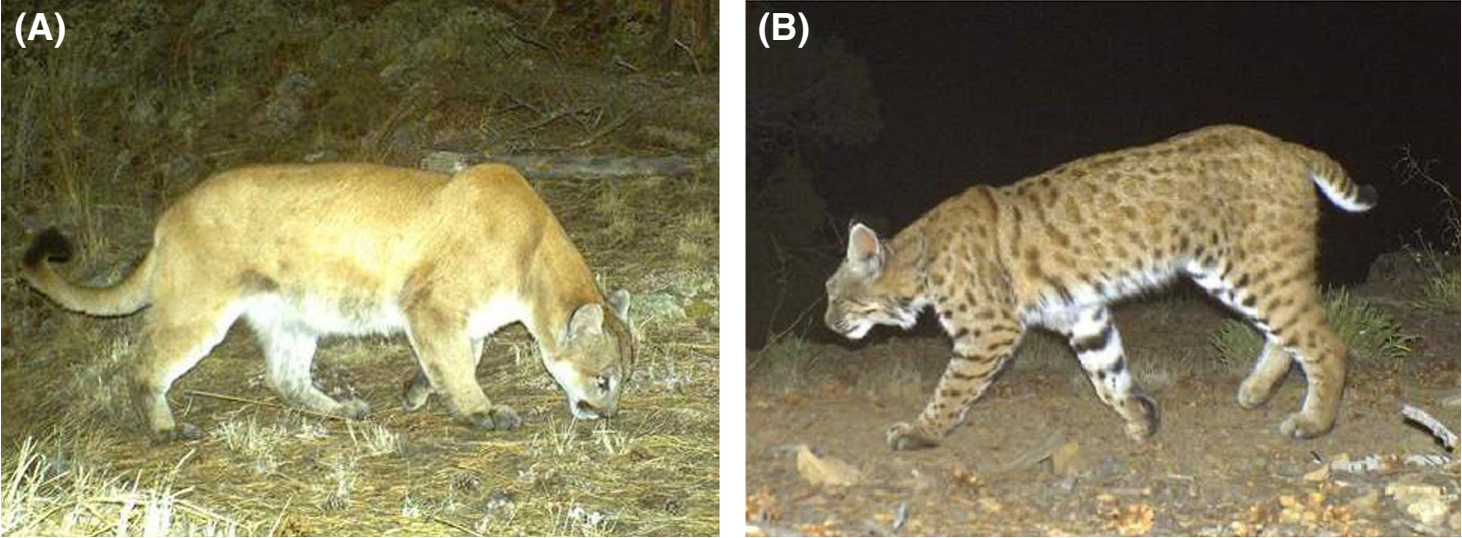
Interspecific interactions between the larger‐bodied puma (A) (typical adult weights range between 40 and 80 kg) and medium‐sized bobcat (B) (typical adult weights range between 7 and 12 kg) were evaluated across multiple levels of urbanization in Colorado, USA. Photographs were obtained from motion‐activated cameras in study areas.

We evaluated interspecific interactions between bobcats and pumas across multiple scales and levels of urbanization. Specifically, we investigated spatial and temporal interactions of bobcats and pumas (Fig. 1) by evaluating circadian activity patterns, broad‐scale seasonal interactions, and fine‐scale daily interactions in WUI, exurban development, and wildland habitat. Overall, we predicted that wild felids would demonstrate greater avoidance in wildland habitat and increased interaction opportunities in urbanized landscapes. Specifically, we predicted high overlap in circadian activity patterns between bobcats and pumas and greater overlap of activity patterns in landscapes impacted by urbanization compared to wildland areas. If bobcats use “competition refuges” in space or time, we expected that bobcats would avoid pumas at both fine and broad scales and hypothesized that interactions would increase in areas associated with urbanization. Further, if bobcats avoid high‐use areas of pumas, we expected a negative relationship between the number of puma observations at a site and detection probability of bobcats.

## Materials and Methods

### Study area

**Figure 2:**
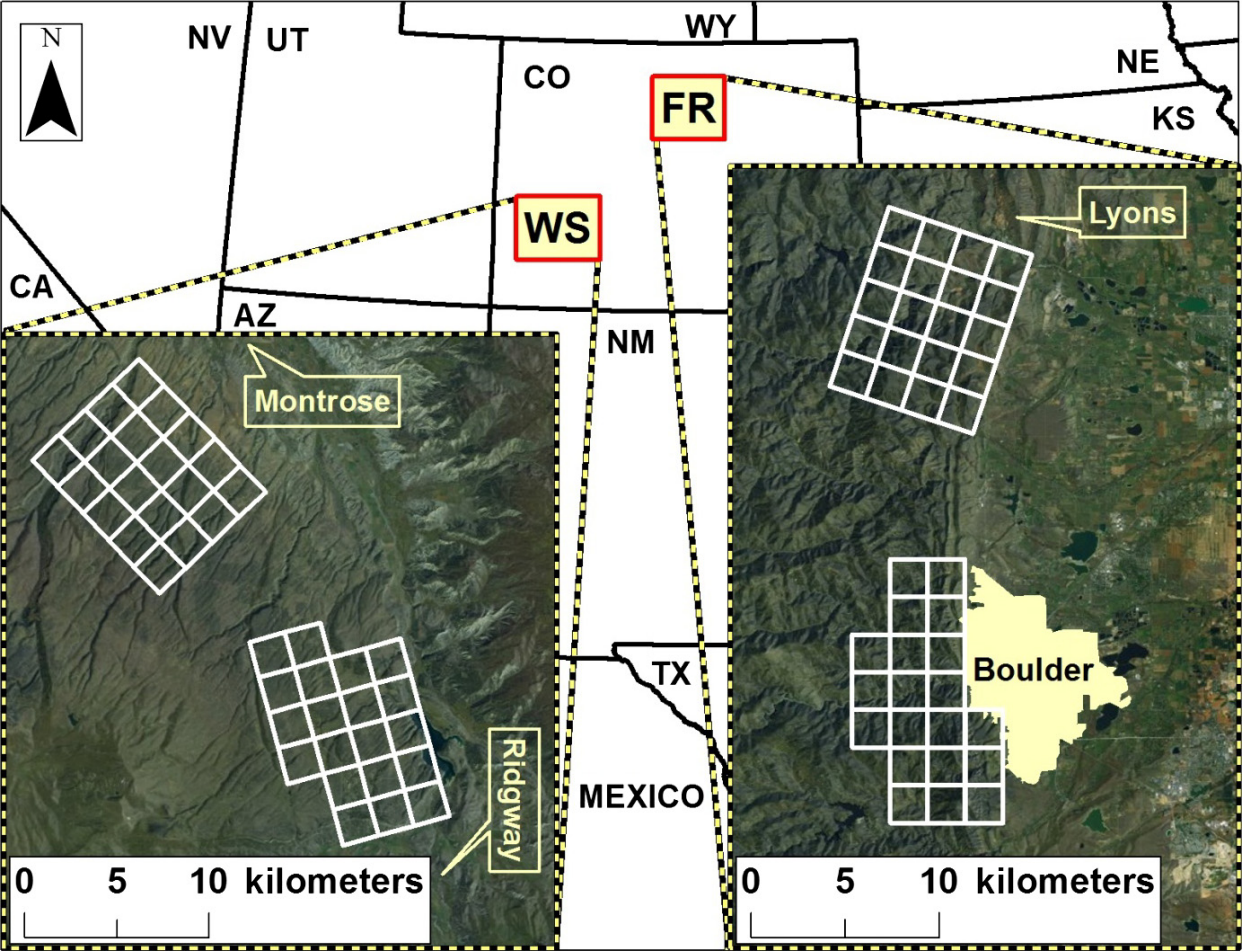
Motion‐activated cameras were maintained across two study sites in Colorado, USA, exhibiting varying levels of urbanization. The more rural Western Slope (WS) was characterized by an exurban development southern grid and a wildland northern grid during 2009. The more urbanized Front Range (FR) study area was characterized by a wildland–urban interface (WUI) southern grid and wildland northern grid during 2010.

We conducted our research across two study areas in Colorado, USA, that exhibited varying degrees of urbanization and human influence. In 2009, we worked on the Western Slope (WS) of Colorado on the Uncompahgre Plateau near the towns of Montrose and Ridgway (Fig. 2). Common vegetation included pinyon pine (*Pinus edulis*) and juniper (*Juniperus osteosperma*), ponderosa pine (*Pinus ponderosa*), aspen (*Populus tremuloides*), Gambel oak (*Quercus gambelii*), and big sagebrush (*Artemisia tridentata*). We divided the WS study area into two sampling grids. The southern grid sampled residential development on Log Hill Mesa (population = 1041; US Census Bureau 2010) that was dominated by exurban and rural development; residential parcel sizes were distributed, from most to least numerous, across 5, 2, 1, ≥5, and ≥40 acre properties. Within areas of residential development, travel corridors of natural habitat and open‐space property, often with associated recreation trails, were present. The northern grid sampled primarily undeveloped, wildland habitat that occurred across public land, although some small areas of private land with low‐density human residences and hunting camps occurred on or near the grid.

In 2010, we worked on the more urbanized Front Range (FR) of Colorado (Fig. 2). Common vegetation included ponderosa pine, Douglas‐fir (*Pseudotsuga menziesii*), juniper, aspen, and mountain mahogany (*Cercocarpus montanus*). A network of open‐space properties with recreational trails occurred across the study area. Similar to the WS, we divided the FR study area into two sampling grids. The southern grid occurred adjacent to the WUI associated with the city of Boulder (population = 97,385, US Census Bureau 2010) and was characterized by open‐space properties with some human residences on or near the grid. The northern grid sampled wildland habitat occurring on public lands, although a small number of rural human residences were present on private property inholdings. See Lewis et al. (2015) for an expanded description of the study area.

### Sampling grids, camera surveys, and sample sizes

Each study area (WS and FR) contained 40 motion‐activated cameras divided between 2 camera grid arrays spaced approximately 6 km apart (Fig. 2). Each grid was 80 km^2^, consisting of 20 2 × 2 km grid cells (the total area sampled was 160 km^2^). Within each grid cell, we placed one motion‐activated camera at a sampling site that we believed maximized the opportunity to photograph bobcats and pumas. Cameras were placed along game trails, people trails, and secondary dirt roads where felid sign (primarily scats, scrapes, and marking sites) was observed or in areas that appeared to be likely travel routes. Each camera was set up approximately 4 m from the travel route in a perpendicular orientation and housed in a metal security box 0.75 m high on a tree or metal post. Our sampling was passive in that we did not use attractants (i.e., sight, sound, scent) to lure animals to the camera location. We used Cuddeback Capture (Non Typical, Inc., Green Bay, WI) motion‐activated cameras (with a 30‐sec delay) with a white flash to obtain color photographs during the day and at night, except at one sampling site along a high‐use human recreation trail on the FR where we switched to using a Cuddeback Attack Infra‐Red camera to reduce vandalism. Cameras operated on the WS from 21 August 2009 to 13 December 2009 and on the FR from 1 October 2010 to 31 December 2010.

We considered photographs of bobcats and pumas taken at a camera site to be independent if images were obtained >1 h apart. If 2 adult felids were photographed <1 h apart and could be differentiated based on natural or artificial (i.e., telemetry collars and eartags; for details see Lewis et al. 2015) markings, these photographs were also counted as independent animals. Kittens and dependent offspring (individuals typically of small body size and often accompanied by their mother in photographs) were not considered independent animals and were excluded from analyses.

During the course of our study, it was estimated that 52.6 (SE = 6.3) bobcats and 14.4 (SE = 1.6) pumas used the WS study area and 55.1 (SE = 11.4) bobcats and 14.7 (SE = 1.3) pumas used the FR study area (Lewis et al. 2015). Thus, these were the number of animals estimated to be available to be sampled across our camera grids.

### Circadian activity patterns

We compared overlap in activity patterns between bobcats and pumas between urbanized and wildland grids (1 comparison per study area). To estimate activity patterns of felids using circular kernel density statistics, we used the R (R Development Core Team 2014) package Overlap (Meredith and Ridout 2013) and followed their recommendations for bandwidth selection, estimators for quantifying overlap, and 10,000 bootstrap simulations to estimate 95% confidence intervals (Ridout and Linkie 2009; Meredith and Ridout 2013). Activity throughout the day was defined as crepuscular (morning and evening), diurnal (day), and nocturnal (night).

### Occupancy modeling

We used occupancy modeling (MacKenzie et al. 2006) to evaluate interactions between bobcats and pumas across broad (seasonal) and fine (daily) scales, described below. Occupancy probability (*Ψ*) estimates the proportion of the landscape used by the species and detection probability (*p*) estimates the probability of detecting a species given that it used a site (i.e., a camera location), which can evaluate the behavioral response in relation to landscape characteristics. A behavioral response assumes that lower detection probability is related to decreased frequency of use due to niche relationships (Royle and Nichols 2003; Richmond et al. 2010; Lewis et al. 2015). All occupancy analyses were conducted in program PRESENCE (Hines 2006) and models were ranked using Akaike information criteria corrected for small sample size (AIC*c*; Burnham and Anderson 2002).

#### Broad scale: seasonal

Competitive interactions can shape species distributions, where dominant species exclude subordinate species from otherwise suitable habitat (Palomares and Caro 1999; Creel et al. 2001; Caro and Stoner 2003). We evaluated the broad‐scale seasonal effect of puma presence on bobcat occupancy and detection using conditional two‐species occupancy models (Richmond et al. 2010) with 5 sampling occasions where each occasion was 22 days long on the WS and 18 days long on the FR. For these models, pumas were the dominant species (species A) and bobcats were the subordinate species (species B). Two‐species occupancy models consider eight parameters related to occupancy and detection probabilities; we focused on five of these parameters to evaluate the model comparisons presented by Richmond et al. (2010) including *Ψ* ^BA^ (probability of occupancy for bobcats, given pumas are present), *Ψ* ^Ba^ (probability of occupancy of bobcats, given pumas are absent), *p* ^B^ (probability of detection for bobcats, given pumas are absent), *r* ^BA^ (probability of detection for bobcats, given both species are present and pumas are detected), and *r* ^Ba^ (probability of detection for bobcats, given both species are present and pumas are not detected). To evaluate whether the occupancy of the subordinate species depends on the presence of the dominant species, we compared the relative performance of models where (1) puma occupancy did not alter bobcat occupancy (*Ψ* ^BA^ = *Ψ* ^Ba^; i.e., the probability of bobcat occupancy of a site is independent of puma occupancy) and (2) puma occupancy altered bobcat occupancy (*Ψ* ^BA^ and *Ψ* ^Ba^ estimated separately; i.e., the probability of bobcat occupancy is different on sites that are used or not used by puma) (Richmond et al. 2010). To evaluate whether bobcat detection probability (i.e., their behavior based on frequency of use) was influenced by puma occupancy, we compared the relative performance of models where (1) pumas did not alter bobcat detection probability (*p* ^B^ = *r* ^B.^, where *r* ^B.^ = (*r* ^BA^ = *r* ^Ba^); i.e., bobcat detection probability was independent of puma use) and (2) pumas altered bobcat detection probability (*p* ^B^ is estimated separately from *r* ^B.^ (*p* ^B^ ≠ *r* ^B.^); i.e., bobcat detection probability was different at sites used and not used by pumas). Some models evaluating *p* ^B^ ≠ *r* ^B.^ failed to converge when estimates of occupancy for the dominant species were approximately 1 (i.e., pumas on the FR; Lewis et al. 2015); when this occurred these models were removed from the model set.

Previous research indicated that landscape covariates did not sufficiently explain occupancy of bobcats and pumas in our study, which was likely due to relatively high estimates of occupancy for felids and little variation in estimated use (Lewis et al. 2015), as well as the scale of analysis. However, detection probabilities of each species were influenced by two covariates: one continuous covariate that measured human influence at each camera site (termed *human development*) and a categorical covariate that characterized each of the sampling grids (termed *grid*) (Lewis et al. 2015). The covariate *human development* measured the amount of human influence (Lewis et al. 2011) associated with each camera location and was created by digitizing each human occurrence point (HOP; residence or structure) in the study areas using ArcMap 10 geographic information system (GIS) software (ESRI, Redlands, CA) from color orthophotographs (Lewis et al. 2015). Using ArcToolBox in ArcMap 10, we fit a Gaussian kernel over each HOP, where the density, or influence, was greatest directly at the point of interest and decreased out to a specified radius of a circle (Lewis et al. 2011); radii ranged from 100–1000 m on the WS and 100–1500 m on the FR (Lewis et al. 2015). In GIS, each camera location was intersected with the cumulative kernel density of human development across each radius (Lewis et al. 2015). To determine which spatial scale of human development was appropriate for each study area, we compared univariate models where detection probability was modeled as a function of the human development covariate across radii and used AIC_*c*_ model ranking to determine the best scale. Based on this approach, we used a radius of 200 m on the WS and 1300 m on the FR (Lewis et al. 2015). The covariate *grid* designated camera sites located in either exurban development or wildland grids (on the WS) or WUI or wildland grids (on the FR). In addition, Lewis et al. (2015) concluded that sampling effort (a time‐varying covariate accounting for the number of days that a camera operated for each sampling occasion) influenced detection probability on the FR, but not the WS; therefore, the covariate *effort* was included for all detection probability parameters on the FR in broad‐scale occupancy analyses. For each broad‐scale model set, we compared models that evaluated how pumas affected bobcat occupancy and how pumas, *human development*, and *grid* affected bobcat detection probability.

#### Fine scale: daily

Scent marking (via scats, urinations, and scent glands) is an important mode of communication among felids, where animals may use olfactory and visual signs to avoid each other in space and time (Gorman and Trowbridge 1989; Logan and Sweanor 2001; Caro and Stoner 2003). We therefore evaluated whether there was a lag effect from 1–4 days in bobcat detection probability after pumas visited a site. That is, will bobcats avoid sites for short periods of time after a puma visit, and did this vary between urbanized and wildland grids? It was hypothesized that bobcats could detect the presence of pumas for up to three additional days once a puma traveled through an area; this number of days was based on the experience of researchers who use trained dogs to track pumas using the scent of animals (K. Logan personal communication).

To evaluate the fine‐scale daily effect of pumas on bobcats, we used single‐species occupancy models (MacKenzie et al. 2002, 2006) to estimate detection probability (behavioral response) of bobcats in relation to covariates. For fine‐scale models, each day represented a sampling occasion (*t* = 113 on the WS and *t* = 92 on the FR) and a species was recorded as detected if at least one photograph was documented between 12:00 (i.e., noon) of consecutive days; this definition of a day was used because of the crepuscular and nocturnal activity patterns of bobcats and pumas. To evaluate whether pumas influenced bobcat detection probability on a fine scale, we created multiple covariates that characterized puma detection at each site for each day *i* (i.e., if a puma was detected [1] or not [0]). First, we created a time‐specific covariate (*P1*) that recorded whether a puma was detected at a site within the 24‐h period (i.e., *t*_*i*_). We then created three covariates (*P2*,* P3*,* P4*) which represented lag effects of puma detection from 1–3 additional days. For example, *P3* represents a covariate that would evaluate whether detection probability of bobcats was different (lower) for 3 days after a puma detection, starting with the day a puma was detected plus the next 2 days (i.e., *P3* covariate would be *t*_*i*_ = 1, *t*_*i*+1_ = 1, *t*_*i*+2_ = 1, when a puma was detected on day *i* at a site). Because we predicted that competitive interactions between bobcats and pumas would be influenced by urbanization (i.e., differ between urbanized and wildland grids), we included interactions between the covariates *grid* and *P1*,* P2*,* P3*, and *P4*. Based on the positive relationship between local abundance and photographic rates (Carbone et al. 2001; Rovero and Marshall 2009) or detection probability (Royle and Nichols 2003; Lewis et al. 2015), we expected a negative relationship between bobcat detection probability and the number of puma photographs recorded at a site during the study because the frequency of bobcat use may decline at sites that are often visited by pumas. We therefore included a site covariate summarizing the total number of puma photographs for each camera location (i.e., *puma count*). We also evaluated the influence of *human development* and *grid* covariates on daily bobcat detection probability (Lewis et al. 2015). As explained above, previous research found that bobcat occupancy in our system was not influenced by covariates, likely due to high estimates of occupancy (Lewis et al. 2015). For each fine‐scale model set, we compared models that evaluated how *human development*,* grid*,* puma count*, puma lag effects (*P1*–*P4*), and the interactions between *grid* and puma lag effects influenced bobcat detection probability.

## Results

We documented a photograph of a felid at each of our camera sites across both study areas, and both species were well represented across sampling grids (Table 1). Based on the proportion of sites that each felid was detected across the WS and FR (Table 1), bobcats and pumas exhibited relatively high values of naïve occupancy (i.e., calculated as the number of sampling sites that a species was detected at divided by the total number of sampling sites for a grid area).

**Table 1:** Summary of photographs for felids in exurban development and wildland habitat on the Western Slope (WS) and in wildland–urban interface (WUI) and wildland habitat on the Front Range (FR) of Colorado, 2009–2010

Study area^a^	Species	Grid area	# Sites^b^	# Photographs
WS	Bobcat	Exurban	20	112
WS	Bobcat	Wildland	18	73
WS	Bobcat	Total	38	185
WS	Puma	Exurban	11	39
WS	Puma	Wildland	12	41
WS	Puma	Total	23	80
FR	Bobcat	WUI	15	81
FR	Bobcat	Wildland	17	69
FR	Bobcat	Total	32	150
FR	Puma	WUI	19	50
FR	Puma	Wildland	17	46
FR	Puma	Total	36	96

a Sampling occurred for 113 days on the WS and 92 days on the FR.

b The number of camera locations (sites) where the species was detected at least once. There were 20 sites on each individual grid.

### Circadian activity patterns

**Figure 3:**
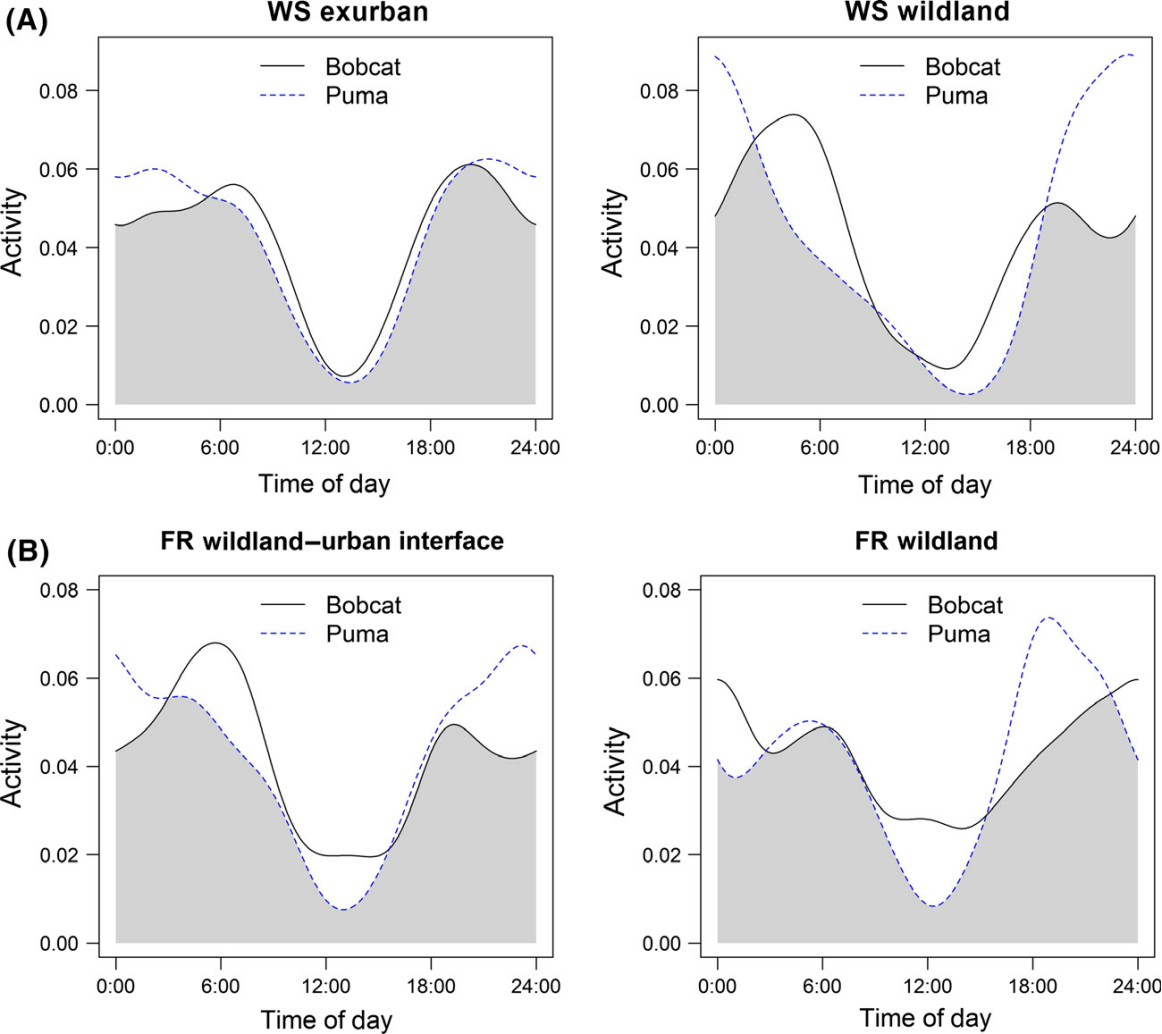
Overlap in activity patterns between bobcats and pumas was greater in exurban development compared to wildland habitat on the Western Slope (WS) during 2009 (A) and similar between wildland–urban interface (WUI) and wildland habitat on the Front Range (FR) during 2010 (B). Kernel density of activity is represented along the *y*‐axis and the 24‐h circadian daily cycle occurs along the *x*‐axis.

As expected, both species were mostly active during crepuscular and nocturnal time periods, with bobcats active more during diurnal time periods than pumas, particularly on the FR (Fig. 3). Puma activity peaked during the evening crepuscular and nocturnal periods, particularly on the WS wildland and both FR grids, while bobcat activity tended to peak during the morning crepuscular and nocturnal periods (Fig. 3). There was more overlap in activity patterns between bobcats and pumas in exurban development compared to wildland habitat on the WS (Table 2; Fig. 3A); 95% confidence intervals between grids (Table 2) overlapped by 16% and proportion overlap between margin of errors equaled 32%. Overlap in activity patterns between felids was similar between WUI and wildland habitat on the FR (Table 2; Fig. 3B); 95% confidence intervals between grids (Table 2) overlapped by 97% and margin of errors overlapped completely.

**Table 2:** Estimated overlap of activity patterns (and associated 95% confidence intervals) between bobcats and pumas in exurban development and wildland habitat on the Western Slope and in wildland–urban interface (WUI) and wildland habitat on the Front Range of Colorado, 2009–2010

Western slope		Front range	
Exurban	Wildland	WUI	Wildland
0.93 (0.86–0.97)	0.77 (0.62–0.89)	0.87 (0.77–0.94)	0.86 (0.76–0.94)

### Occupancy

#### Broad scale: seasonal

At the broad seasonal scale, the presence of pumas did not appear to exclude bobcats from sites or decrease their detection. For both the WS and FR, models that indicated bobcat occupancy and detection probability were similar in the presence and absence of pumas were always more supported based on AIC_*c*_ values than models where bobcat occupancy and/or detection probability varied based on puma presence at a site (Tables 3 and 4). On the WS, the top model reported that (1) estimates of occupancy of bobcats when pumas were absent and present were similar (*Ψ* ^Ba^ = *Ψ* ^BA^ = 0.99, SE = 0.04), (2) detection probability of bobcats when pumas were absent and present was similar within the exurban development (*p* ^B^ = *r* ^B.^ = 0.61, SE = 0.06) and wildland (*p* ^B^ = *r* ^B.^ = 0.44, SE = 0.07) grids, and (3) occupancy of pumas (*Ψ* ^A^) was 0.61 (SE = 0.08). Similarly, on the FR, the top model reported that (1) estimates of occupancy for bobcats when pumas were absent and present were similar (*Ψ* ^Ba^ = *Ψ* ^BA^ = 0.83, SE = 0.06), (2) detection probability of bobcats when pumas were absent and present was similar within the WUI (*p* ^B^ = *r* ^B.^ = 0.54, SE = 0.09) and wildland (*p* ^B^ = *r* ^B.^ = 0.46, SE = 0.07) grids, and (3) occupancy of pumas was high (*Ψ* ^A^ = 0.99, SE = 0.06). Consistent with Lewis et al. (2015), the covariates *human development* and *grid* improved model performance (Tables 3 and 4). For the parameter *p* ^B^ = *r* ^B.^ in the top model, covariate estimates demonstrated a negative relationship of bobcat detection probability with *human development* (WS: *β *= −0.38, SE = 0.17; FR: *β *= −0.56, SE = 0.22) and *grid* (WS: *β *= −0.79, SE = 0.33; FR: *β *= −0.83, SE = 0.38). Based on model comparisons at the broad seasonal scale, bobcat behavior appeared to be more influenced by the covariates *human development* and *grid*, than the presence of pumas.

**Table 3:** Model selection results for broad‐scale 2‐species occupancy models evaluating seasonal interactions between bobcats and pumas on the Western Slope, Colorado, 2009. Parameters included *Ψ* ^A^ (probability of occupancy for pumas), *Ψ* ^BA^ (probability of occupancy for bobcats, given pumas are present), *Ψ* ^Ba^ (probability of occupancy of bobcats, given pumas are absent), *p* ^A^ (probability of detection for pumas, given bobcats are absent), *r* ^A^ (probability of detection for pumas, given both species are present), *p* ^B^ (probability of detection for bobcats, given pumas are absent), *r* ^BA^ (probability of detection for bobcats, given both species are present and pumas are detected), and *r* ^Ba^ (probability of detection for bobcats, given both species are present and pumas are not detected). Covariates included: G (sampling grid area) and HD (influence of human development at a kernel density radius of 200 m)

Model^a^	*K*	AIC_*c*_	ΔAIC_*c*_	*ω*	log (L)
*Ψ* ^A^, (*Ψ* ^BA^ = *Ψ* ^Ba^), *p* ^A^, *r* ^A^, (*p* ^B^ = *r* ^BA^ = *r* ^Ba^(G + HD))	7	480.67	0.00	0.45	466.67
*Ψ* ^A^, *Ψ* ^BA^, *Ψ* ^Ba^, *p* ^A^, *r* ^A^, (*p* ^B^ = *r* ^BA^ = *r* ^Ba^(G + HD))	8	481.66	0.99	0.27	465.66
*Ψ* ^A^, (*Ψ* ^BA^ = *Ψ* ^Ba^), *p* ^A^, *r* ^A^, (*p* ^B^ = *r* ^BA^ = *r* ^Ba^(G))	6	484.37	3.70	0.07	472.37
*Ψ* ^A^, (*Ψ* ^BA^ = *Ψ* ^Ba^), *p* ^A^, *r* ^A^, (*p* ^B^ = *r* ^BA^ = *r* ^Ba^(HD))	6	484.49	3.82	0.07	472.49
*Ψ* ^A^, *Ψ* ^BA^, *Ψ* ^Ba^, *p* ^A^, *r* ^A^, (*p* ^B^ = *r* ^BA^ = *r* ^Ba^(HD))	7	484.77	4.10	0.06	470.77
*Ψ* ^A^, *Ψ* ^BA^, *Ψ* ^Ba^, *p* ^A^, *r* ^A^, (*p* ^B^ = *r* ^BA^ = *r* ^Ba^(G))	7	485.59	4.92	0.04	471.59
*Ψ* ^A^, (*Ψ* ^BA^ = *Ψ* ^Ba^), *p* ^A^, *r* ^A^, *p* ^B^(G + HD), (*r* ^BA^ = *r* ^Ba^(G + HD))	10	485.62	4.95	0.04	465.62
*Ψ* ^A^, *Ψ* ^BA^, *Ψ* ^Ba^, *p* ^A^, *r* ^A^, *p* ^B^(G + HD), (*r* ^BA^ = *r* ^Ba^(G + HD))	11	486.87	6.20	0.02	464.87
*Ψ* ^A^, (*Ψ* ^BA^ = *Ψ* ^Ba^), *p* ^A^, *r* ^A^, (*p* ^B^ = *r* ^BA^ = *r* ^Ba^)	5	487.10	6.43	0.02	477.10
*Ψ* ^A^, *Ψ* ^BA^, *Ψ* ^Ba^, *p* ^A^, *r* ^A^, (*p* ^B^ = *r* ^BA^ = *r* ^Ba^)	6	487.53	6.86	0.01	475.53
*Ψ* ^A^, (*Ψ* ^BA^ = *Ψ* ^Ba^), *p* ^A^, *r* ^A^, *p* ^B^(G), (*r* ^BA^ = *r* ^Ba^(G))	8	488.29	7.62	0.01	472.29
*Ψ* ^A^, *Ψ* ^BA^, *Ψ* ^Ba^, *p* ^A^, *r* ^A^, *p* ^B^(HD), (*r* ^BA^ = *r* ^Ba^(HD))	9	488.49	7.82	0.01	470.49
*Ψ* ^A^, (*Ψ* ^BA^ = *Ψ* ^Ba^), *p* ^A^, *r* ^A^, *p* ^B^, (*r* ^BA^ = *r* ^Ba^)	6	489.10	8.43	0.01	477.10
*Ψ* ^A^, *Ψ* ^BA^, *Ψ* ^Ba^, *p* ^A^, *r* ^A^, *p* ^B^, (*r* ^BA^ = *r* ^Ba^)	7	489.51	8.84	0.00	475.51
*Ψ* ^A^, *Ψ* ^BA^, *Ψ* ^Ba^, *p* ^A^, *r* ^A^, *p* ^B^(G), (*r* ^BA^ = *r* ^Ba^(G))	9	489.54	8.87	0.00	475.54
*Ψ* ^A^, (*Ψ* ^BA^ = *Ψ* ^Ba^), *p* ^A^, *r* ^A^, *p* ^B^, *r* ^BA^, *r* ^Ba^	7	490.94	10.27	0.00	476.94
*Ψ* ^A^, (*Ψ* ^BA^ = *Ψ* ^Ba^), *p* ^A^(HD), *r* ^A^(HD), *p* ^B^(HD), *r* ^BA^(HD), *r* ^Ba^(HD)	12	491.05	10.38	0.00	467.05
*Ψ* ^A^, *Ψ* ^BA^, *Ψ* ^Ba^, *p* ^A^, *r* ^A^, *p* ^B^, *r* ^BA^, *r* ^Ba^	8	491.31	10.64	0.00	475.31
*Ψ* ^A^, *Ψ* ^BA^, *Ψ* ^Ba^, *p* ^A^(HD), *r* ^A^(HD), *p* ^B^(HD), *r* ^BA^(HD), *r* ^Ba^(HD)	13	491.50	10.83	0.00	465.50
*Ψ* ^A^, (*Ψ* ^BA^ = *Ψ* ^Ba^), *p* ^A^(G + HD), *r* ^A^(G + HD), *p* ^B^(G + HD), *r* ^BA^(G + HD), *r* ^Ba^(G + HD)	17	492.62	11.95	0.00	458.62
*Ψ* ^A^, (*Ψ* ^BA^ = *Ψ* ^Ba^), *p* ^A^(G), *r* ^A^(G), *p* ^B^(G), *r* ^BA^(G), *r* ^Ba^(G)	12	492.73	12.06	0.00	468.73
*Ψ* ^A^, *Ψ* ^BA^,*Ψ* ^Ba^, *p* ^A^(G), *r* ^A^(G), *p* ^B^(G), (*r* ^BA^ = *r* ^Ba^ (G))	12	492.75	12.08	0.00	468.75
*Ψ* ^A^, *Ψ* ^BA^, *Ψ* ^Ba^, *p* ^A^(G + HD), *r* ^A^(G + HD), *p* ^B^(G + HD), *r* ^BA^(G + HD), *r* ^Ba^(G + HD)	18	493.20	12.53	0.00	457.20
*Ψ* ^A^, (*Ψ* ^BA^ = *Ψ* ^Ba^), *p* ^A^, *r* ^A^, *p* ^B^(HD), (*r* ^BA^ = *r* ^Ba^(HD))	8	494.57	13.90	0.00	478.57
*Ψ* ^A^, *Ψ* ^BA^, *Ψ* ^Ba^, *p* ^A^(G), *r* ^A^(G), *p* ^B^(G), *r* ^BA^(G), *r* ^Ba^(G)	13	495.72	15.05	0.00	469.72

a To evaluate whether the occupancy of bobcats depends on the presence of pumas, we compared conditional occupancy models (*Ψ* ^BA^ and *Ψ* ^Ba^ estimated separately) to unconditional models (*Ψ* ^BA^ = *Ψ* ^Ba^). To evaluate whether the detection of bobcats was influenced by the presence of pumas, we compared conditional detection models (*p* ^B^ is estimated separately from *r* ^BA^ and *r* ^Ba^, assuming *r* ^BA^ = *r* ^Ba^) to unconditional models (*p* ^B^ = *r* ^BA^ = *r* ^Ba^) (Richmond et al. 2010).

**Table 4:** Model selection results for broad‐scale 2‐species occupancy models evaluating seasonal interactions between bobcats and pumas on the Front Range, Colorado, 2010. Parameters included *Ψ* ^A^ (probability of occupancy for pumas), *Ψ* ^BA^ (probability of occupancy for bobcats, given pumas are present), *Ψ* ^Ba^ (probability of occupancy of bobcats, given pumas are absent), *p* ^A^ (probability of detection for pumas, given bobcats are absent), *r* ^A^ (probability of detection for pumas, given both species are present), *p* ^B^ (probability of detection for bobcats, given pumas are absent), *r* ^BA^ (probability of detection for bobcats, given both species are present and pumas are detected), and *r* ^Ba^ (probability of detection for bobcats, given both species are present and pumas are not detected). Covariates included: G (sampling grid area), HD (influence of human development at a kernel density radius of 1300 m), and E (sampling effort)

Model^a^	*K*	AIC_*c*_	ΔAIC_*c*_	*ω*	log (L)
*Ψ* ^A^, (*Ψ* ^BA^ = *Ψ* ^Ba^), *p* ^A^(E), *r* ^A^(E), (*p* ^B^ = *r* ^BA^ = *r* ^Ba^(G + HD + E))	10	508.60	0.00	0.46	488.60
*Ψ* ^A^, *Ψ* ^BA^, *Ψ* ^Ba^, *p* ^A^(E), *r* ^A^(E), (*p* ^B^ = *r* ^BA^ = *r* ^Ba^(G + HD + E))	11	510.60	2.00	0.17	488.60
*Ψ* ^A^, (*Ψ* ^BA^ = *Ψ* ^Ba^), *p* ^A^(E), *r* ^A^(E), (*p* ^B^ = *r* ^BA^ = *r* ^Ba^(HD + E))	9	511.28	2.68	0.12	493.28
*Ψ* ^A^, *Ψ* ^BA^, *Ψ* ^Ba^, *p* ^A^(E), *r* ^A^(E), (*p* ^B^ = *r* ^BA^ = *r* ^Ba^(HD + E))	10	513.20	4.60	0.05	493.20
*Ψ* ^A^, (*Ψ* ^BA^ = *Ψ* ^Ba^), *p* ^A^(E), *r* ^A^(E), (*p* ^B^ = *r* ^BA^ = *r* ^Ba^(E))	8	513.38	4.78	0.04	497.38
*Ψ* ^A^, (*Ψ* ^BA^ = *Ψ* ^Ba^), *p* ^A^(E), *r* ^A^(E), (*p* ^B^ = *r* ^BA^ = *r* ^Ba^(G + E))	9	514.00	5.40	0.03	496.00
*Ψ* ^A^, (*Ψ* ^BA^ = *Ψ* ^Ba^), *p* ^A^(G + HD + E), *r* ^A^(G + HD + E), *p* ^B^(G + HD + E), *r* ^BA^(G + HD + E), *r* ^Ba^(G + HD + E)	22	514.47	5.87	0.02	470.47
*Ψ* ^A^, (*Ψ* ^BA^ = *Ψ* ^Ba^), p^A^(E), r^A^(E), p^B^(HD + E), (r^BA^ = r^Ba^(HD + E))	12	514.95	6.35	0.02	490.95
*Ψ* ^A^, *Ψ* ^BA^, *Ψ* ^Ba^, *p* ^A^(E), *r* ^A^(E), (*p* ^B^ = *r* ^BA^ = *r* ^Ba^(E))	9	515.36	6.76	0.02	497.36
*Ψ* ^A^, (*Ψ* ^BA^ = *Ψ* ^Ba^), *p* ^A^(E), *r* ^A^(E), *p* ^B^(G + E), (*r* ^BA^ = *r* ^Ba^(G + E))	12	515.44	6.84	0.02	491.44
*Ψ* ^A^, *Ψ* ^BA^, *Ψ* ^Ba^, *p* ^A^(E), *r* ^A^(E), (*p* ^B^ = *r* ^BA^ = *r* ^Ba^(G + E))	10	516.00	7.40	0.01	496.00
*Ψ* ^A^, (*Ψ* ^BA^ = *Ψ* ^Ba^), *p* ^A^(E), *r* ^A^(E), *p* ^B^(E), (*r* ^BA^ = *r* ^Ba^(E))	10	516.25	7.65	0.01	496.25
*Ψ* ^A^, *Ψ* ^BA^, *Ψ* ^Ba^, *p* ^A^(G + HD + E), *r* ^A^(G + HD + E), *p* ^B^(G + HD + E), *r* ^BA^(G + HD + E), *r* ^Ba^(G + HD + E)	23	516.47	7.87	0.01	470.47
*Ψ* ^A^, *Ψ* ^BA^, *Ψ* ^Ba^, *p* ^A^(E), *r* ^A^(E), *p* ^B^(HD + E), (*r* ^BA^ = *r* ^Ba^(HD + E))	13	516.95	8.35	0.01	490.95
*Ψ* ^A^, *Ψ* ^BA^, *Ψ* ^Ba^, *p* ^A^(E), *r* ^A^(E), *p* ^B^(G + E), (*r* ^BA^ = *r* ^Ba^(G + E))	13	517.29	8.69	0.01	491.29
*Ψ* ^A^, *Ψ* ^BA^, *Ψ* ^Ba^, *p* ^A^(E), *r* ^A^(E), *p* ^B^(E), (*r* ^BA^ = *r* ^Ba^(E))	11	518.24	9.64	0.00	496.24
*Ψ* ^A^, (*Ψ* ^BA^ = *Ψ* ^Ba^), *p* ^A^(HD + E), *r* ^A^(HD + E), *p* ^B^(HD + E), *r* ^BA^(HD + E), *r* ^Ba^(HD + E)	17	518.72	10.12	0.00	484.72
*Ψ* ^A^, (*Ψ* ^BA^ = *Ψ* ^Ba^), *p* ^A^(E), *r* ^A^(E), *p* ^B^(E), *r* ^BA^(E), *r* ^Ba^(E)	12	519.78	11.18	0.00	495.78
*Ψ* ^A^, *Ψ* ^BA^, *Ψ* ^Ba^, *p* ^A^(G + E), *r* ^A^(G + E), *p* ^B^(G + E), (*r* ^BA^ = *r* ^Ba^(G + E))	15	520.51	11.91	0.00	490.51
*Ψ* ^A^, *Ψ* ^BA^, *Ψ* ^Ba^, *p* ^A^(HD + E), *r* ^A^(HD + E), *p* ^B^(HD + E), *r* ^BA^(HD + E), *r* ^Ba^(HD + E)	18	520.70	12.10	0.00	484.70
*Ψ* ^A^, *Ψ* ^BA^, *Ψ* ^Ba^, *p* ^A^(E), *r* ^A^(E), *p* ^B^(E), *r* ^BA^(E), *r* ^Ba^(E)	13	521.85	13.25	0.00	495.85
*Ψ* ^A^, (*Ψ* ^BA^ = *Ψ* ^Ba^), *p* ^A^(G + E), *r* ^A^(G + E), *p* ^B^(G + E), *r* ^BA^(G + E), *r* ^Ba^(G + E)	17	522.25	13.65	0.00	488.25
*Ψ* ^A^, *Ψ* ^BA^, *Ψ* ^Ba^, *p* ^A^(G + E), *r* ^A^(G + E), *p* ^B^(G + E), *r* ^BA^(G + E), *r* ^Ba^(G + E)	18	525.03	16.43	0.00	489.03

a To evaluate whether the occupancy of bobcats depends on the presence of pumas, we compared conditional occupancy models (*Ψ* ^BA^ and *Ψ* ^Ba^ estimated separately) to unconditional models (*Ψ* ^BA^ = *Ψ* ^Ba^). To evaluate whether the detection of bobcats was influenced by the presence of pumas, we compared conditional detection models (*p* ^B^ is estimated separately from *r* ^BA^ and *r* ^Ba^, assuming *r* ^BA^ = *r* ^Ba^) to unconditional models (*p* ^B^ = *r* ^BA^ = *r* ^Ba^) (Richmond et al. 2010).

#### Fine scale: daily

**Figure 4:**
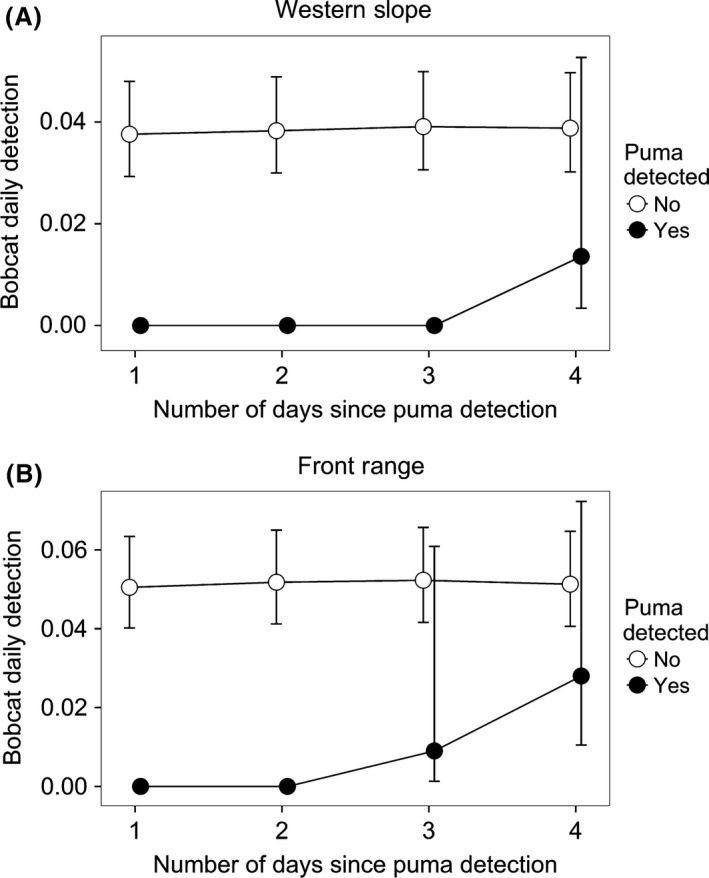
Bobcat daily detection probability estimates (with associated 95% confidence intervals) were lower for 2–3 days after a puma visited a site in wildland habitat on the Western Slope (A) and Front Range (B) of Colorado. Bobcat detection probability was evaluated in relation to 1‐ to 4‐day lag periods of puma detection at a site considering the interaction between *grid* (urbanized or wildland) and each lag effect of puma detection (*P1* to *P4*) on bobcat detection probability using single‐species occupancy models.

**Figure 5:**
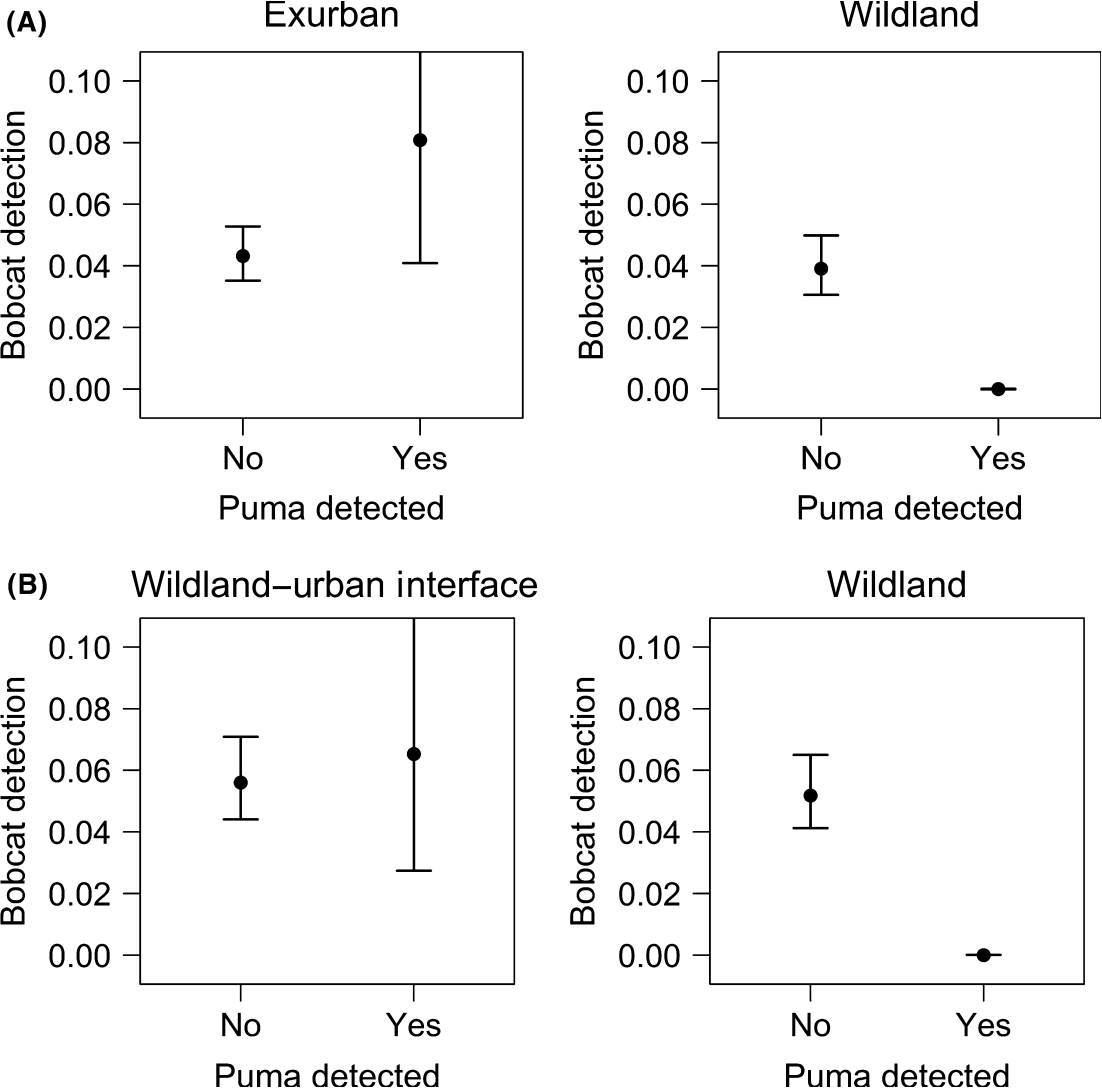
Bobcat daily detection probability estimates (with associated 95% confidence intervals) in relation to puma detection in exurban development and wildland habitat on the Western Slope (WS) in 2009 (A) and wildland–urban interface (WUI) and wildland habitat on the Front Range (FR) in 2010 (B). Estimates are based on the interaction between *grid* and puma lag effect of 3 days on the WS (A) and the interaction between *grid* and puma lag effect of 2 days on the FR (B) using single‐species occupancy models.

At the fine daily scale, puma detection explained temporal and spatial variation in bobcat detection probability in both study areas. Temporally, pumas appeared to affect the detection probability of bobcats for relatively short periods of time (i.e., up to a few days); however, results varied across landscapes experiencing different levels of urbanization. On the WS and FR, all the top models included an interaction between the detection of pumas (*P1*–*P4* covariates) and sampling grids (*grid* covariate) for bobcat detection probability (Tables 5 and 6). The best model for the WS contained an interaction between *grid* and the lag effect of pumas on bobcat detection probability, which lasted up to 3 days (Table 5). On the wildland grid, daily detection probability of bobcats remained at zero for 3 days after puma detection and then increased toward levels observed when pumas were not detected at a site (Fig. 4A). Thus, WS bobcats were less likely to be detected for short periods of time after pumas visited a site on the wildland grid; however, this pattern was not observed on the exurban development grid (Fig. 5A), indicating that bobcats did not avoid pumas for short periods of time in this type of urbanized habitat. On the FR, the most supported model indicated that bobcat detection probability was lower on the wildland grid when pumas were detected, but only up to 2 days after a puma visited a site (Table 6). Bobcat detection probability remained at 0 for 2 days and then increased at 3 and 4 days after a puma visit to a site (Fig. 4B). However, detection probability of bobcats was similar on the FR WUI grid when pumas were present and absent (Fig. 5B), again suggesting that bobcats did not avoid pumas on fine scales in landscapes influenced by urbanization.

**Table 5:** Model selection results for fine‐scale single‐species single‐season occupancy models for bobcats evaluating daily interactions with pumas on the Western Slope, Colorado, 2009. Parameters included *Ψ* (occupancy; probability of use for bobcats) and *p* (detection probability for bobcats). Covariates included PumaCount (total number of independent puma photographs recorded at a camera site), HD (influence of human development at a kernel density radius of 200 m), G (sampling grid area), *P*1 (same‐day detection of puma, no additional lag effect), *P*2 (day of puma detection plus 1 additional day of lag effect), *P*3 (day of puma detection plus 2 additional days of lag effect), *P*4 (day of puma detection plus 3 additional days of lag effect), G*P (interaction term between sampling grid area and the lag effect of puma detection from 1–4 days)

Model	*K*	AIC_*c*_	ΔAIC_*c*_	*ω*	log (L)
*Ψ*(.), *p*(PumaCount + HD + G + *P*3 + G*P3)	7	1426.06	0.00	0.52	1412.06
*Ψ*(.), *p*(PumaCount + HD + G + *P*2 + G*P2)	7	1426.98	0.92	0.33	1412.98
*Ψ*(.), *p*(PumaCount + HD + G + *P*4 + G*P4)	7	1430.27	4.21	0.06	1416.27
*Ψ*(.), *p*(G + *P*3 + G*P3)	5	1432.36	6.30	0.02	1422.36
*Ψ*(.), *p*(PumaCount + HD + G + *P*1 + G*P1)	7	1433.21	7.15	0.01	1419.21
*Ψ*(.), *p*(PumaCount + HD)	4	1433.32	7.26	0.01	1425.32
*Ψ*(.), *p*(G + *P*2 + G*P2)	5	1433.84	7.78	0.01	1423.84
*Ψ*(.), *p*(PumaCount + HD + *P*2)	5	1434.61	8.55	0.01	1424.61
*Ψ*(.), *p*(PumaCount + HD + *P*4)	5	1434.68	8.62	0.01	1424.68
*Ψ*(.), *p*(PumaCount + HD + *P*1)	5	1435.26	9.20	0.01	1425.26
*Ψ*(.), *p*(PumaCount + HD + *P*3)	5	1435.28	9.22	0.01	1425.28
*Ψ*(.), *p*(HD)	3	1435.60	9.54	0.00	1429.60
*Ψ*(.), *p*(G + *P*4 + G*P4)	5	1437.08	11.02	0.00	1427.08
*Ψ*(.), *p*(PumaCount)	3	1438.72	12.66	0.00	1432.72
*Ψ*(.), *p*(.)	2	1439.11	13.05	0.00	1435.11
*Ψ*(.), *p*(G)	3	1439.55	13.49	0.00	1433.55
*Ψ*(.), *p*(G + *P*1 + G*P1)	5	1439.79	13.73	0.00	1429.79
*Ψ*(.), *p*(*P*2)	3	1440.90	14.84	0.00	1434.90
*Ψ*(.), *p*(*P*4)	3	1441.03	14.97	0.00	1435.03
*Ψ*(.), *p*(*P*3)	3	1441.06	15.00	0.00	1435.06
*Ψ*(.), *p*(*P*1)	3	1441.11	15.05	0.00	1435.11

**Table 6:** Model selection results for fine‐scale single‐species single‐season occupancy models for bobcats evaluating daily interactions with pumas on the Front Range, Colorado, 2010. Parameters included *Ψ* (occupancy; probability of use for bobcats) and *p* (detection probability for bobcats). Covariates included PumaCount (total number of independent puma photographs recorded at a camera site), HD (influence of human development at a kernel density radius of 1300 m), G (sampling grid area), *P*1 (same‐day detection of puma, no additional lag effect), *P*2 (day of puma detection plus 1 additional day of lag effect), *P*3 (day of puma detection plus 2 additional days of lag effect), *P*4 (day of puma detection plus 3 additional days of lag effect), G*P (interaction term between sampling grid area and the lag effect of puma detection from 1–4 days)

Model	*K*	AIC_*c*_	ΔAIC_*c*_	*ω*	log (L)
*Ψ*(.), *p*(PumaCount + HD + G + *P*2 + G*P2)	7	1176.81	0.00	0.45	1162.81
*Ψ*(.), *p*(PumaCount + HD + G + *P*3 + G*P3)	7	1179.01	2.20	0.15	1165.01
*Ψ*(.), *p*(PumaCount + HD + G + *P*1 + G*P1)	7	1179.15	2.34	0.14	1165.15
*Ψ*(.), *p*(G + *P*2 + G*P2)	5	1181.16	4.35	0.05	1171.16
*Ψ*(.), *p*(PumaCount + HD)	4	1182.27	5.46	0.03	1174.27
*Ψ*(.), *p*(PumaCount + HD + G + *P*4 + G*P4)	7	1182.89	6.08	0.02	1168.89
*Ψ*(.), *p*(PumaCount + HD + *P*2)	5	1183.18	6.37	0.02	1173.18
*Ψ*(.), *p*(G + *P*3 + G*P3)	5	1183.31	6.50	0.02	1173.31
*Ψ*(.), *p*(PumaCount + HD + *P*3)	5	1183.37	6.56	0.02	1173.37
*Ψ*(.), *p*(PumaCount)	3	1183.91	7.10	0.01	1177.91
*Ψ*(.), *p*(G + *P*1 + G*P1)	5	1184.01	7.20	0.01	1174.01
*Ψ*(.), *p*(HD)	3	1184.03	7.22	0.01	1178.03
*Ψ*(.), *p*(.)	2	1184.20	7.39	0.01	1180.20
*Ψ*(.), *p*(PumaCount + HD + *P*4)	5	1184.21	7.40	0.01	1174.21
*Ψ*(.), *p*(PumaCount + HD + *P*1)	5	1184.27	7.46	0.01	1174.27
*Ψ*(.), *p*(*P*2)	3	1184.62	7.81	0.01	1178.62
*Ψ*(.), *p*(*P*3)	3	1184.76	7.95	0.01	1178.76
*Ψ*(.), *p*(G)	3	1185.46	8.65	0.01	1179.46
*Ψ*(.), *p*(*P*4)	3	1185.91	9.10	0.00	1179.91
*Ψ*(.), *p*(*P*1)	3	1186.16	9.35	0.00	1180.16
*Ψ*(.), *p*(G + *P*4 + G*P4)	5	1187.54	10.73	0.00	1177.54

**Figure 6:**
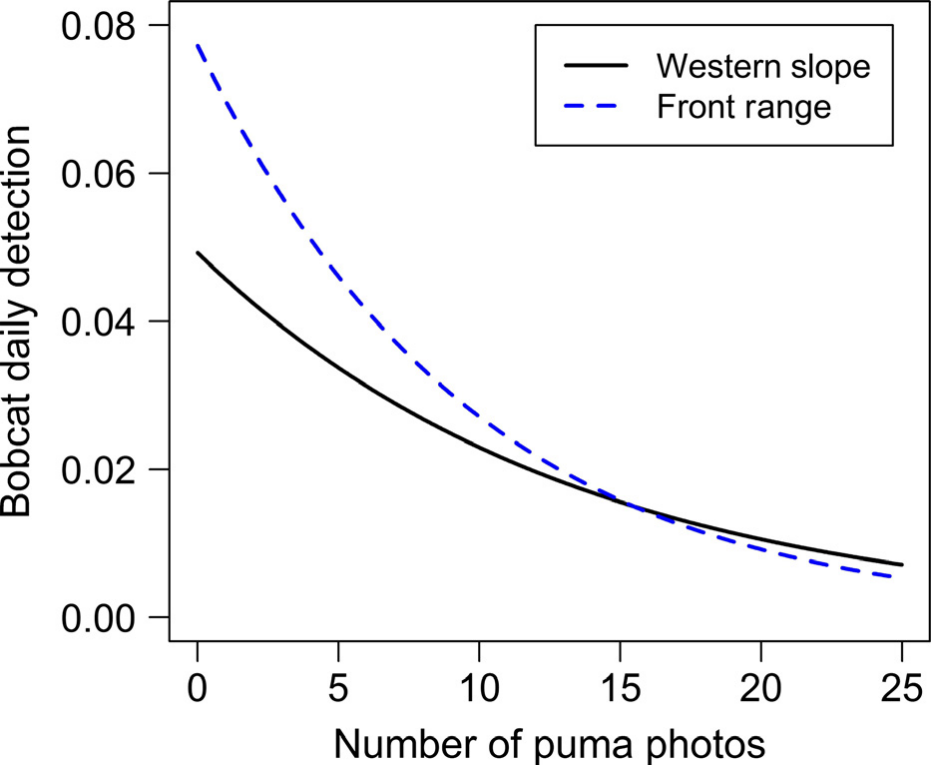
The effect of pumas on daily detection probability of bobcats varied by puma use at a site. Bobcat detection probability decreased with the number of puma photographs at a camera location (i.e., *puma count*) on the Western Slope (WS) and Front Range (FR) of Colorado. Parameter estimates from the top models (Tables 5 and 6) were used to plot the relationship for *puma count*,* human development* (mean value), *grid* 1, *puma lag effect* (=0), and *puma lag effect* interaction (=0). Our data for the number of puma photographs at a site ranged from 0–8 images over 113 days on the WS and from 0–6 images over 92 days on the FR. Results are extrapolated to visualize the predicted response of bobcat detection probability in relation to higher frequencies of puma detections at a site.

The number of puma detections at a site also influenced bobcat detection probability. Based on the top models, *puma count* demonstrated a negative relationship with bobcat detection probability on the WS (*β *= −0.08; SE = 0.04) and FR (*β *= −0.11; SE = 0.05). Therefore, as the number of puma photographs at a camera site increased, the probability of detecting bobcats decreased (Fig. 6). The number of puma photographs recorded at a camera site ranged from 0–8 on the WS and from 0–6 on the FR. Consistent with broad‐scale results above, the covariate *human development* improved model performance and demonstrated a negative relationship with bobcat detection probability on both WS and FR.

## Discussion

Consistent with our predictions, urbanization altered the opportunity for wild felids to interact. A dominant carnivore did not exclude a subordinate carnivore across broad spatial and temporal scales. However, bobcats responded behaviorally to the presence of pumas at finer scales, and such avoidance patterns varied across levels of urbanization. In wildland habitat, bobcats avoided using areas for short temporal periods (i.e., 2–3 days) once a puma visited an area, but then used these sites with similar probability after approximately 4 days compared to sites where pumas were not recently detected. Bobcats likely detected the presence of pumas through markings and scent along trails and responded by altering their behavior to avoid direct interactions with a superior competitor. In contrast to wildland habitat, in landscapes influenced by urbanization (exurban development and WUI habitat) bobcats did not avoid areas that pumas recently visited. In addition, in low‐density exurban development, overlap in circadian activity patterns between bobcats and pumas increased compared to wildland habitat. Thus, urbanization can potentially lead to increased opportunities for interspecific competition with potential far‐reaching impacts to felid populations and the ecological community.

Population densities of animals might increase in urbanized habitat due to greater landscape heterogeneity and food (Chace and Walsh 2006), restricted dispersal due to anthropogenic barriers (Riley et al. 2006), or ecological release from competitors (Crooks and Soulé 1999), which can increase opportunities for interspecific interactions (Crooks et al. 2010). Our results indicated that the opportunity for interactions between felids increased in habitat influenced by urban development; however, this pattern did not appear to be related to increased population densities of felids. In our study areas, population densities for both bobcats and pumas were lower in exurban development compared to wildland habitat and densities were similar between WUI and wildland habitat (Lewis et al. 2015). We thus consider three additional hypotheses for how interspecific interactions could increase in areas influenced by urbanization.

First, increased overlap in activity patterns could result in animals being more active during similar times. Animals may shape their circadian activity patterns in response to interspecific competition and are thus able to reduce the opportunity for interference competition with competitors by being active during different times of the day (Carothers and Jaksić 1984; Kronfeld‐Schor and Dayan 2003). However, in urbanized areas, animals may shift their activity patterns to avoid human disturbance, thus increasing overlap in activity patterns and the potential for direct interactions. For example, if animals avoid human disturbance during the day and find temporal refuge from human activities at night (e.g., George and Crooks 2006), then animals might be active during restricted temporal periods, which can potentially lead to increased interaction opportunities. On the WS, anthropogenic disturbance likely altered circadian activity patterns of felids in exurban development, where animals were more active at night to avoid human disturbance during the day, leading to greater temporal overlap. On the FR, although we did not observe greater overlap in activity patterns between felids on WUI and wildland grids, it is possible that human disturbance emanating from urban areas could alter activity patterns of animals in other systems, such as in smaller patches of habitat surrounded by an urban matrix, or at finer spatial scales along the WUI. Further, human recreation, which can alter activity patterns in animals (George and Crooks 2006), occurred across the FR and might have influenced activity of felids similarly between grids.

Second, by altering landscape pattern or increasing landscape fragmentation, animal movements might be funneled to avoid human disturbance, and this could result in animals being more likely to use similar areas. Thus, decreased movement options across the landscape could elevate the use of shared habitat and increase the opportunity for interactions. In addition, carnivores often use human recreation trails and dirt roads as travel routes, which can influence animal movement behavior (Karanth et al. 2010). If animals are more likely to use well‐defined trails created by humans as travel routes, as is often the case with wild felids, animal movements, and thus potential interactions, might be more concentrated in these areas as well. On the WS and FR, animals were likely funneled into using more restrictive areas of natural habitat because they avoided areas of human development and disturbance, but used travel corridors and natural habitat that were intermixed or adjacent to residential development.

Third, scent marking disturbance can occur from human activities, which could increase interaction opportunities if animals are unable to detect one another. Scent marking through scats, urinations, and scent glands is an important and widespread form of communication among animals (Ralls 1971; Wyatt 2014), especially within carnivore communities (Gorman and Trowbridge 1989; Logan and Sweanor 2001; Sunquist and Sunquist 2002). In our study, results of fine‐scale interactions in wildland habitat indicate that subordinate carnivores detect and avoid dominant carnivores via scent. Scent marking often occurs in prominent locations along trails to advertise the presence of animals, which can either be territorial (e.g., warning other animals of an individual's presence) or as an advertisement (e.g., providing information about the mating status of animals) (Wyatt 2014). Human activities, however, can destroy or mask such scent marking signals and thus disrupt communication among animals or lead animals to increase scent marking activities. For example, along trails used by humans, this can occur through recreationists trampling and destroying animal scent marks or domestic dogs ingesting carnivore scats (coprophagy; Soave and Brand 1991; Boze 2010) or urinating or defecating at marking sites (Bekoff 2001). The introduction of novel scents and markings from domestic dogs can also increase the use of trails by some wildlife to investigate and remark sites (Lenth et al. 2008). Thus, due to scent marking disturbance, animals might be less aware of each other's presence or more active on human trails, leading to increased interspecific interactions. On the FR, high levels of human recreation on trails associated with the WUI (Vaske et al. 2009) might have disturbed carnivore markings and scent and thus disrupted the ability of animals to detect conspecifics.

In addition to temporal avoidance, bobcats also appeared to avoid pumas spatially. The probability of detecting bobcats decreased as the number of puma visits increased at a site, suggesting that bobcats less frequently used areas with high visitation rates by pumas. If the number of puma visits to a site was exceptionally high (indicating a strong preference for an area), then bobcat detection probability could approach zero. However, we caution that detection probabilities of zero do not necessarily imply nonuse of a site by animals (MacKenzie et al. 2006). In our study, puma visits to a site were relatively low; but in other systems, it would be predicted that areas with high use by a dominant competitor could potentially exclude subordinate species. For example, other research indicates that high‐use areas by top carnivores can influence the frequency of use by subordinate competitors, potentially leading to exclusion of the subordinate species from such areas (e.g., Durant 1998; Creel et al. 2001).

The spatial and temporal avoidance exhibited by bobcats in response to pumas is consistent with a behavioral strategy of a subordinate carnivore to reduce the potential for aggressive competitive interactions and interspecific killing. Two bobcats were likely killed by pumas at deer carcasses in our study (one in exurban development and one in wildland habitat; Lewis personal observation) and interspecific killing between these felids has been reported elsewhere (e.g., Young 1978; Koehler and Hornocker 1991); however, other studies where these species overlap have not reported incidences of pumas killing bobcats (e.g., Knick 1990; Logan and Sweanor 2001). Although such occurrences appear to be uncommon in wildland habitat, long‐term research is necessary to understand how varying levels of urbanization affect the frequency of aggressive interactions and rates of mortality from interspecific competition.

Additional factors could influence interspecific interactions that either we did not evaluate or could be more pronounced in other ecological systems. For example, urbanization can influence the population densities of a variety of competitors, which can alter ecological communities and competitive interactions (Crooks and Soulé 1999; Faeth et al. 2005; Crooks et al. 2010). Estimates of population density were not available for other potential competitors in our study, such as red fox (*Vulpes vulpes*), gray fox (*Urocyon cinereoargenteus*), coyotes (*Canis latrans*), and black bears (*Ursus americanus*); however, population densities for these species can increase in urban‐associated areas (Beckmann and Berger 2003; Gehrt et al. 2010), which could possibly influence space‐use patterns and interactions among felids. Prey populations, such as small mammals and ungulates, also can potentially be influenced by urbanization (Bolger et al. 1997; Polfus and Krausman 2012; Riem et al. 2012); although it is unknown to what degree prey influenced interspecific interactions in our system, estimates of occupancy for key prey species were similar within study areas (Lewis et al. 2015), suggesting that availability of prey did not affect interspecific interactions between wild felids. However, prey populations can influence the distribution and interactions of carnivores in other systems (Robinson et al. 2014). In addition, seasonal and annual variation in landscape pattern and populations of competitors and prey can alter the strength of competitive interactions through time (Wiens 1977; Schoener 1982).

The conservation implications of our study are that the conversion of wildland habitat to urbanization will likely alter interactions among species and potentially affect animal populations and community structure. For example, we observed greater opportunities for encounters between bobcats and pumas in urbanized environments, which could lead to higher rates of aggressive interspecific contact and interspecific killing and subsequently increased transmission rates of pathogens in urban areas (Franklin et al. 2007). Further, by potentially funneling animal movements into more restrictive travel corridors, there could be increased opportunities for incidences with people and domestic animals in such areas. Ultimately, multiple mechanisms, as proposed above, can alter competition in urbanized habitat, and such mechanisms might vary depending upon the form and intensity of urbanization. These considerations can be incorporated into land‐use planning to minimize impacts to wildlife communities and reduce potential interactions with people. Our findings suggest that by managing for wildland habitat and reducing human disturbance in such areas, animals will likely be better able to maintain spatial and temporal separation to reduce the potential of competitive interactions.

## Conflict of Interest

None declared.
